# Impact of Number of Segmented Tissues on SAR Prediction Accuracy in Deep Pelvic Hyperthermia Treatment Planning

**DOI:** 10.3390/cancers12092646

**Published:** 2020-09-16

**Authors:** Iva VilasBoas-Ribeiro, Gerard C. van Rhoon, Tomas Drizdal, Martine Franckena, Margarethus M. Paulides

**Affiliations:** 1 Department of Radiation Oncology, Erasmus MC Cancer Institute, 3015 GD Rotterdam, The Netherlands; g.c.vanrhoon@erasmusmc.nl (G.C.v.R.); m.franckena@erasmusmc.nl (M.F.); m.m.paulides@tue.nl (M.M.P.); 2 Department of Radiation Science and Technology, Faculty of Applied Sciences, Delft University of Technology, 2629 JB Delft, The Netherlands; 3 Department of Biomedical Technology, Czech Technical University in Prague, nam. Sitna 3105, 272 01 Kladno, Czech Republic; tomas.drizdal@fbmi.cvut.cz; 4 Electromagnetics for Care & Cure (EM-4C&C) Laboratory, Center for Care and Cure Technologies Eindhoven (C3Te), Department of Electrical Engineering, Eindhoven University of Technology, 5600 MB Eindhoven, The Netherlands

**Keywords:** RF hyperthermia, deep hyperthermia treatment planning, 3D patient modeling, tissue delineation and segmentation

## Abstract

**Simple Summary:**

Hyperthermia treatment planning is the process of optimizing treatment quality using pre-treatment simulations. Although it has become a powerful tool, prediction accuracy is strongly dependent on the patient model. For deep hyperthermia in the pelvis, it is common that only four tissue categories are discriminated (bone, fat, muscle-like, and tumor). For the head and neck region, more tissues have been shown to be required for good prediction accuracy. Delineating is a labor-intensive and difficult process. Hence, it is important to find the optimum between accuracy and labor, but for deep pelvic hyperthermia, there are no published studies showing the impact of the number of tissues. We studied the trade-off between the segmentation detail needed and segmentation feasibility. Our findings indicate that including high water content tissues can impact simulation accuracy. Although our results, in general, underline the suitability of our current clinical protocol, they help to prioritize improvements for specific cases.

**Abstract:**

In hyperthermia, the general opinion is that pre-treatment optimization of treatment settings requires a patient-specific model. For deep pelvic hyperthermia treatment planning (HTP), tissue models comprising four tissue categories are currently discriminated. For head and neck HTP, we found that more tissues are required for increasing accuracy. In this work, we evaluated the impact of the number of segmented tissues on the predicted specific absorption rate (SAR) for the pelvic region. Highly detailed anatomical models of five healthy volunteers were selected from a virtual database. For each model, seven lists with varying levels of segmentation detail were defined and used as an input for a modeling study. SAR changes were quantified using the change in target-to-hotspot-quotient and maximum SAR relative differences, with respect to the most detailed patient model. The main finding of this study was that the inclusion of high water content tissues in the segmentation may result in a clinically relevant impact on the SAR distribution and on the predicted hyperthermia treatment quality when considering our pre-established thresholds. In general, our results underline the current clinical segmentation protocol and help to prioritize any improvements.

## 1. Introduction

During hyperthermia treatments, tumor temperature is elevated towards 40 to 44 °C to sensitize tumor cells for chemo- and/or radiotherapy [1,2,3,4]. Several clinical studies have shown that the addition of hyperthermia to radiotherapy results in improved clinical outcome, without added toxicity [5,6,7,8,9]. A retrospective analysis by Franckena et al. [10] showed that the probability of cure is positively correlated with the administered thermal dose delivered in the target based on intraluminal temperature measurements in 420 patients [11], which was confirmed recently by Kroesen et al. [12] in a new patient cohort and state-of-the-art radiotherapy. In the last decades, hyperthermia treatment planning (HTP) has been considered a powerful tool for optimizing treatment quality [9,13,14,15]. HTP is either used to optimize the specific absorption rate (SAR) distribution as a surrogate for the thermal dose [16,17,18] or to optimize the temperature distribution [19,20]. The importance of HTP is also illustrated by the decision of the European Society on Hyperthermic Oncology (ESHO) to include HTP in their quality assurance guidelines for deep hyperthermia [15,21,22,23]. HTP prediction accuracy is, however, strongly dependent on the patient model [24,25,26,27]. For head and neck hyperthermia, the study by Verhaart et al. showed many tissues are required for an accurate and realistic representation [28]. For deep loco-regional hyperthermia in the pelvic region, to our knowledge, there are no published studies showing this need, and the impact of the number of tissues that are segmented is unknown. Schooneveldt et al. [27] showed that the bladder content cannot be represented as a muscle-like tissue and should be modeled as a fluid. Hence, SAR will be somewhat higher due to the higher conductivity of urine versus muscle. The greatest impact, however, is expected for thermal modeling based HTP, where thermal washout will be much greater.

At the Erasmus MC Cancer Institute, HTP generally consists of the following steps: (1) obtaining patient data (preferably in treatment position), (2) segmentation of four major tissue types on computed tomography (CT) scans using auto segmentation based on the difference in Hounsfield units (HU), (3) generation of the 3D patient model in the hyperthermia applicator, and (4) calculation and optimization of power deposited in the tissues [29,30]. Due to the strongly variable and patient-specific cooling, our focus on HTP in clinical practice is to improve the energy deposition as a surrogate for the thermal dose [31]. Clinical studies have demonstrated good correlation between calculated temperature changes and simulated changes in SAR [16,31,32,33]. The modeling study performed by Canters et al. [32] showed that the calculated T50 (median target temperature) is highly correlated with the change in target–hotspot–quotient (THQ). This theoretical study also indicates the suitability of THQ as a surrogate for clinical outcome, but this finding needs validation in clinical practice.

Earlier, it has been shown that the 3D anatomical models’ level of detail derived from the segmentation directly affects the recommended amplitude and phase settings of the antennas [18,24,34,35]. The manual segmentation of several tissues from CT data is labor-intensive and requires many staff-hours. Due to the time-consuming process and inability to accurately delineate certain tissues, there is a strong need to study the influence of tissue segmentation on the predicted SAR distribution [36]. This influence may also indirectly impact the thermal dose [10,12,16,32]. As mentioned previously, in our current clinical practice, 3D patient pelvic models are created by segmenting four major tissue types based on the difference in Hounsfield units (HU): bone, muscle-like (muscle plus organs), fat, and gastrointestinal air [37]. For an optimal result, there is a need to know how detailed the characterization of the patient anatomies should be to capture all relevant tissues and tissues transitions with high contrast in the electrical properties, i.e., permittivity and conductivity [24,35,38]. The optimum trade-off between segmentation detail and related labor is currently unknown, and also segmentation feasibility (contrast between tissue types on the CT images or magnetic resonance (MR) images) has to be taken into consideration. Hence, a definitive study to investigate the required segmentation detail is needed.

Therefore, in this study, we investigated the impact of tissue segmentation detail on hyperthermia treatment quality prediction and hyperthermia treatment quality accuracy in a group of highly detailed representative pre-processed human body models. The impact of the number of segmented tissues on the predicted SAR was evaluated using a set of seven tissue segmentation lists that vary in detail (number of tissues). Two aspects were taken into account to select which tissues should be included: (a) visibility of boundaries on the currently used imaging modality (CT) and (b) dielectric contrast with adjacent tissues. By investigating the volume percentage of fat and muscle, we verified that the models from volunteers represent the patients treated in our clinic well. To properly reflect our clinical situation, different realistically shaped cancer types were added to the selected models from healthy volunteers. Next, we studied the impact of segmentation detail on (1) the SAR distribution and (2) the predicted hyperthermia treatment quality. The relation between segmentation detail and simulation accuracy was studied using the original (most detailed) models as a ground-truth. Hence, this study was done in a relative manner, where the only variables that were changed were the delineated tissues.

## 2. Materials and Methods

### 2.1. Patient Models Selection 

**Figure 1:**
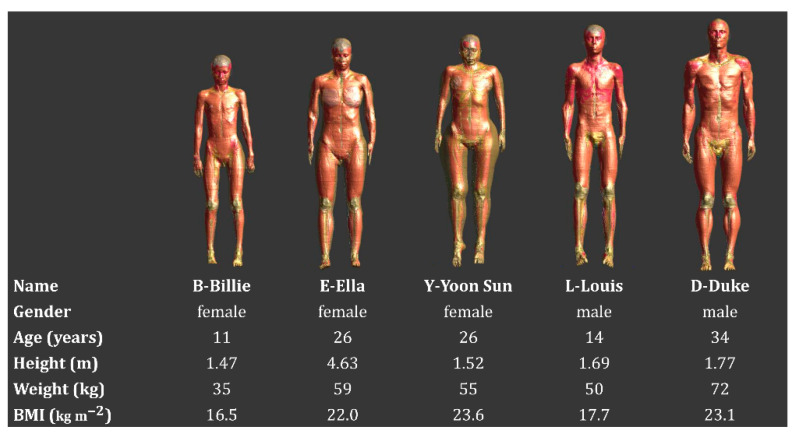
Representation of the virtual family models used (Billie, Ella, Yoon Sun, Louis, and Duke) together with the characteristics of each model (age, height, weight, and BMI: body mass index).

Five anatomical models were selected that matched our patients in terms of gender, fat/muscle percentage, weight, and height. These models were developed at the IT’IS Foundation and belong to the “Virtual Family” [39,40]. Figure 1 shows the five models used in this study and their characteristics. The virtual models used in this study include approximately 80 tissues and are posable, so model posture can be changed to represent realistic scenarios. All models were “posed” to match the position of patients during treatment, i.e., the arms are crossed.

To investigate the representative value of the models in this study, 109 clinical patient models created for regular treatment planning were analyzed to investigate the percentage of fat and muscle. Models of patients treated in the MR-deep hyperthermia applicator (MR-compatible Sigma-Eye) and the non-compatible MR-deep hyperthermia applicator (Sigma-Eye and Sigma-60) were included. Since a typical CT for HTP purposes is performed from the lower part of the sternum down to the patient’s knees, the percentage was calculated only for the patient volume inside the hyperthermia applicator.

**Figure 2:**
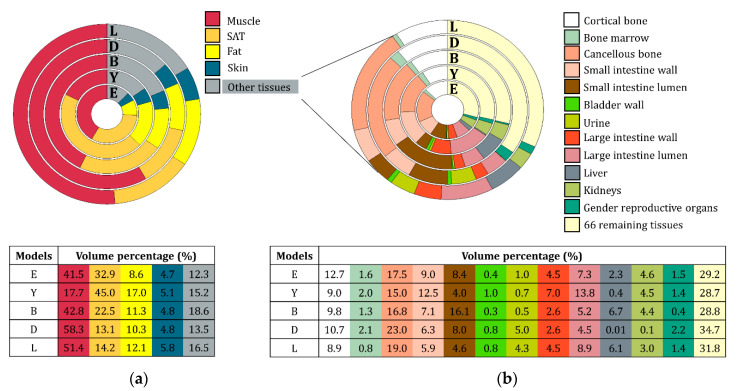
Tissue volume percentage in each virtual model for the body part enclosed by the hyperthermia applicator. The letters presented in the tables and charts correspond to the virtual models: E-Ella; Y-Yoon Sun; B-Billie; D-Duke; L-Louis. (**a**) represents the total tissue volume percentage in each volume, where only the high percentage volume tissues are presented: muscle, fat, SAT (subcutaneous fat), skin, and the remaining other tissues; (**b**) represents the detailed volume percentage of approximately 79 tissues included in the remaining other tissues of (**a**).

The volume of certain organs/tissues varies considerably between the virtual models. Figure 2a presents the percentage of muscle, SAT (saturated fat) assumed as subcutaneous fat and fat skin, and the remaining other tissues. The tissues illustrated in the chart are the ones that present a higher volume percentage. Figure 2b shows the volume percentage of the remaining tissues, where the total number of tissues presented was approximately 79 tissues. The second chart takes into account only the other tissues not mentioned in the previous chart Figure 2a. The table below each pie chart presents the tissue percentage volume of the corresponding virtual model.

### 2.2. Tissue Segmentation Lists 

**Figure 3:**
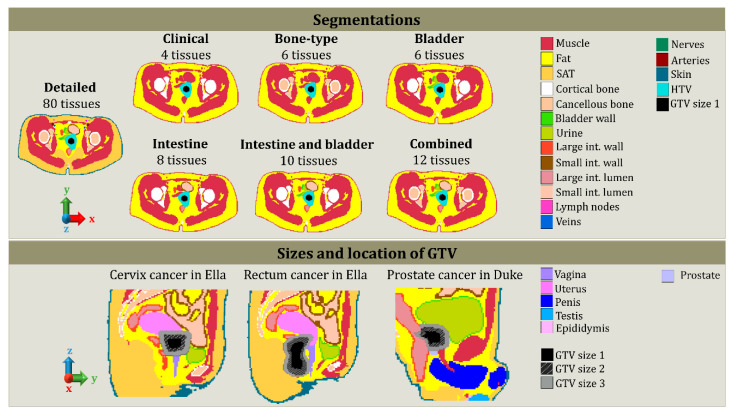
Schematic representation of the different segmentations, sizes, and locations of the GTV (gross target volume). Each color represents a delineated tissue. The virtual model used for displaying the differences between segmentations is Ella. The location and size of GTV are illustrated in Ella and Duke. As presented in the different segmentations, HTV was always larger than the GTV, since it contains the GTV plus a margin. For all the GTV, approximately the same margin was applied to create HTV (hyperthermia target volume). This way, each GTV has a corresponding HTV.

In clinical practice, the segmentation is generally done for four major tissue types: bone, high-water content tissues, low-water content tissues, and gastrointestinal air. The high-water content tissues present the dielectric properties of muscle, and low-water content tissues are assigned with the dielectric properties of fat [38]. Figure 3 illustrates the seven segmentation tissue lists used in this study, and Table 1 describes the tissues delineated in these seven segmentation lists. The choice of the segmentation was made based on tissue contrast and how feasible it is, i.e., visible on CT images, to segment the tissue in CT images, and tissues that can have an impact on the energy deposition due to the dielectric properties.

The most frequently treated tumors are cervical cancer and rectum cancer in females and prostate and rectum cancer in males. Hence, for each patient model, a tumor was defined and added under the supervision of a Radiation Oncologist specialized in Hyperthermia (MF). Two structures were defined: the gross target volume (GTV) and the hyperthermia target volume (HTV). Clinically, the GTV for cervix and prostate cancer consists of the cervix and prostate itself, respectively, and for rectal cancer, GTV encompasses the rectosigmoid. The HTV is the target volume covering GTV and a margin to account for microscopic extensions and local spread. These structures are presented in Figure 3, where a schematic representation of cervical, prostate, and rectum cancer is found. Duke and Ella, from the virtual population, were the male and female anatomical models used to illustrate the location and shape of GTV and HTV. To account for all treated tumors, for each type, the size was modified. Therefore, for each tumor, we varied the GTV to 3 different sizes, as presented in Figure 3, and for each GTV, a corresponding HTV was drawn.

During hyperthermia treatment at Erasmus MC, a transurethral catheter is positioned inside the patient’s bladder, which is kept open during the hyperthermia treatment, so an empty bladder is assumed. The virtual family population is based on anatomic models where the bladder is full, and its content presents urine dielectric properties. Hereto, the impact of urine in the bladder was studied. In the other segmentation lists, the urine is assigned to muscle-equivalent dielectric properties. Additionally, subcutaneous adipose tissue (SAT) is assumed equivalent to visceral fat.

### 2.3. SAR-Based Hyperthermia Treatment Planning

Electromagnetic propagation in the 3D patient models was predicted using the finite-difference time-domain (FDTD) solver in Sim4Life (v.4.4 Zurich MedTech AG, Zurich, Switzerland). The 3D patient model, together with the hyperthermia applicator model, Sigma-Eye were imported in Sim4life. A non-uniform grid was used in the simulations: maximum grid step of 2.5 mm inside the applicator and maximum 10 mm outside the applicator. The total number of voxels per simulation was between 20.9 M (Billy)–23.8 M (Duke) cells. An absorbing boundary condition was selected at the boundaries of the computational domain and 15 periods of the harmonic signal at 100 MHz were necessary to achieve a steady state. Electromagnetic field (EM) distributions were calculated individually for each of 12 antennas for a frequency of 100 MHz. The resulting 3D EM field distributions were imported into the treatment planning software, VEDO, i.e., a custom-made tool developed at Erasmus MC [41].

Table 2 lists the dielectric properties of the delineated tissues in the different segmentations at 100 MHz. In both virtual models, air was not considered. With the exception of the target, all dielectric properties were taken from the IT’IS database, tumor tissue average values were used as are reported in [42,43].

The SAR distribution was optimized to maximize THQ (target to hotspot quotient) using the particle-swarm-based optimizer in VEDO [30]. The total combined electric fields of all antennas were optimized by adapting the complex weights, i.e., the amplitude (power) and phases of these fields, to maximize the SAR within the hyperthermia target volume (HTV) and minimize the SAR in the hotspots. The goal function used was the clinical THQ [30,32], which is formulated as (1):

(1) $$\mathrm{THQ}\;=\;\frac{{\mathrm{SAR}}_{\mathrm{HTV}}}{{\mathrm{SAR}}_{V0.1}}$$

THQ is defined as the ratio between the mean SAR in the target area (${\mathrm{SAR}}_{\mathrm{HTV}}$) and the mean SAR in the hotspots, i.e., the 1% volume of healthy tissue with the highest SAR outside the target region (${\mathrm{SAR}}_{V0.1}$).

The benchmark segmentation, i.e., “detailed”, was used to optimize the antenna settings for each tumor position, and these settings were applied when simulating the SAR for all other (less detailed) patient models.

### 2.4. Dosimetry Evaluation

The evaluation of the simulated SAR distributions was done using THQ as the standard synthetic HTP parameter [32,44]. For each segmentation, the relative difference of maximum average SAR was evaluated in each tissue in comparison to the detailed segmentation. This way, the impact of delineating the tissues, presented in Table 2, was quantified on tissue dose for all the tissues in the model.

Multiple clinical studies have demonstrated a relationship between measured thermal dose expressed as temperature or CEM43 and treatment outcome [10,12,45,46,47,48]. Therefore, for this analysis, we used the correlation found by Canters et al. [32] to define which tissues have a higher impact on the simulated T50 change. This theoretical study defined a decrease of 0.2 °C in simulated T50 as a clinically relevant decrease with respect to treatment outcome, and that this decrease was correlated with a change of 5% in THQ.

The detailed segmentation is considered to be the benchmark, i.e., the ground truth. The absolute change in THQ (|dTHQ|) was computed using the relative difference between the THQ of the detailed reference model with the THQ calculated for the other models. The formulation of the hyperthermia-treatment quality parameter is given by Equation (2).

(2) $$\left|\mathrm{dTHQ}|\;(\%\right)\;=\;\left|\;\left(\frac{{\mathrm{THQ}}_{n}\;-\;{\mathrm{THQ}}_{\mathrm{reference}}}{{\mathrm{THQ}}_{\mathrm{reference}}}\right)\times 100\;\right|$$

where ${\mathrm{THQ}}_{n}$ is the target–hotspot–quotient acquired using the segmentation n, which can be the clinical, bone, intestine, bladder and combined segmentation and ${\mathrm{THQ}}_{\mathrm{reference}}$ is the THQ for the detailed segmentation.

In addition, we analyzed the difference between the maximum average SAR of each tissue, between the less detailed segmentation and the detailed benchmark. This allowed us to quantitatively express the impact of segmentation in the local power deposition for all different tissues. The absolute relative difference (|dRD|.) was calculated using (3):

(3) $$\left|\mathrm{dRD}\right|.{\;}_{\mathrm{tissue}}\;(\%)\;=\;\left|\frac{{\mathrm{Maximum}\;\mathrm{SAR}}_{\mathrm{tissue},\;n}{-\mathrm{Maximum}\;\mathrm{SAR}}_{\mathrm{tissue},\;\mathrm{reference}}}{{\mathrm{Maximum}\;\mathrm{SAR}}_{\mathrm{tissue},\;\mathrm{reference}}}\times 100\right|$$

where ${\mathrm{Maximum}\;\mathrm{SAR}}_{\mathrm{tissue},\;n}$ is the maximum average SAR over 1% of a tissue volume using segmentation n. ${\mathrm{Maximum}\;\mathrm{SAR}}_{\mathrm{tissue},\;\mathrm{reference}}$ is the maximum average SAR over 1% of tissue volume using the detailed segmentation. A threshold between 0% and 20% was set as a reasonable maximum |dRD|. between the benchmark and the other segmentation lists.

## 3. Results

### 3.1. Representative Value of the Virtual Family Models

**Figure 4:**
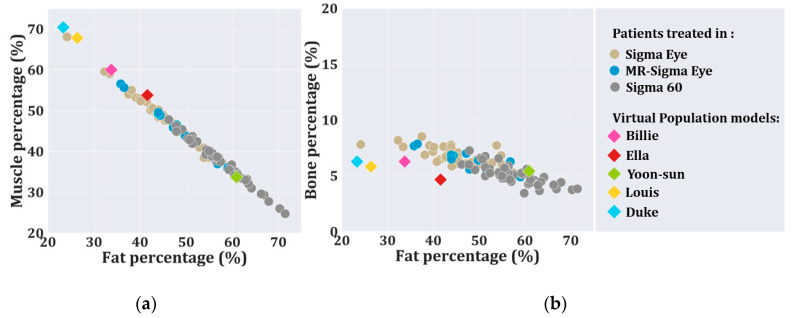
Scatter plots of muscle and bone volume percentage as a function of fat volume percentage, in patients treated and the virtual population models. (**a**) presents the fat and muscle percentage of the treated patients in deep-hyperthermia applicators. (**b**) presents the fat and bone percentage of the treated patients in the hyperthermia applicators mentioned. The fat, muscle, and bone percentage of the virtual models are placed with larger marker size.

Figure 4 presents the fat and muscle percentage of 109 treated patients in deep hyperthermia and the virtual models used in this study by different colored markers. The average and standard deviation of muscle, fat, and bone percentage in the total treated patients was 42.4 ± 7.5%, 51.7 ± 8.4%, and 5.9 ± 1.1%, respectively. For the virtual population models, the average and standard deviation of muscle, fat, and bone percentage was 57.1 ± 14.7%, 37.2 ± 15.0%, and 5.7 ± 0.7%. The muscle and fat percentages were highly correlated (R^2^ = 0.99). Figure 4a shows that the virtual models followed the same regression line as the patient population. The bone percentage followed the same trend between the patient population and the virtual models. For patients and virtual models, the standard variation of the bone percentage was lower than the standard variation of fat and muscle. Figure 4b shows that the bone percentage was not as well correlated with fat (R^2^ = 0.61).

### 3.2. Impact of Tissue Segmentation on Treatment Quality (|dTHQ|)

Table 3 and Table 4 present |dTHQ| obtained for each model and according to the studied segmentation groups. Again |dTHQ| is the difference in THQ of the model compared to the detailed segmentation as the benchmark. As stated, for each model, three different GTV and HTV sizes were taken into account. For each tumor and model, the maximum THQ change was taken into account. Therefore, between the three GTV sizes, only the model that presented the highest variation was included in the analysis.

For female models, in both cancer types, most (31/36) of the |dTHQ| comparisons were larger than the 5% threshold indicating clinical relevance. The highest |dTHQ| was obtained for the bladder segmentation applied to Billie and cervix cancer, i.e., 23.8%. Adding the segmentation of bladder and bones to the basic clinical segmentation did not aid in improving |dTHQ|. Inclusion of the intestines did diminish |dTHQ|, making the (intestine and combined segmentation) model an acceptable representation. This pattern was much less pronounced for the Yoon Sun and Billie model. Note that the volume percentage of intestines in female models was higher than the other segmented tissues, i.e., 29%, 37%, and 31%, respectively in Ella, Yoon Sun, and Billie. For the Ella model, it was not possible to select a segmentation list that kept |dTHQ| below 5% for both cervix and rectum cancer.

In males, introducing the bladder structure into the clinical segmentation was sufficient, since in all models, the |dTHQ| was always lower than 5%.

### 3.3. Impact of Tissue Segmentation on SAR Prediction (|dRD|)

**Figure 5:**
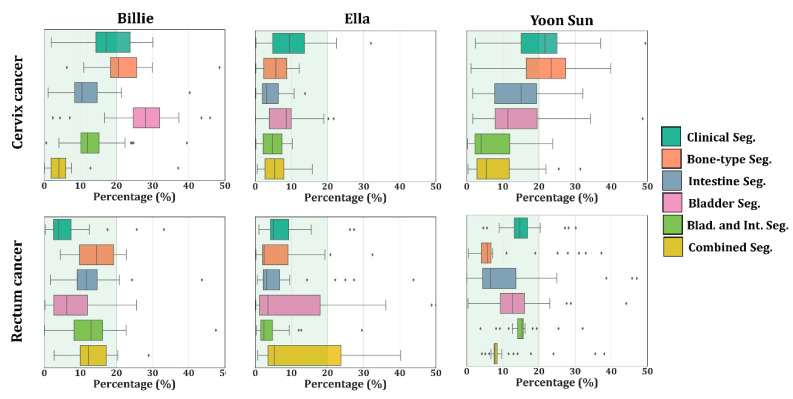
Boxplot with the maximum |dRD| in the female models. |dRD| was normalized to the maximum SAR for each tissue in the benchmark and expressed as a percentage (%). The inter-quartile range represents the middle 50% of the dataset, where the right box line represents 75% of the dataset that falls below the upper quartile, the left line represents 25% of the dataset that falls below the lower quartile, and finally, the middle line represents the median. The line extending from the box represents 2.5% and 97.5% limits of the dataset.

**Figure 6:**
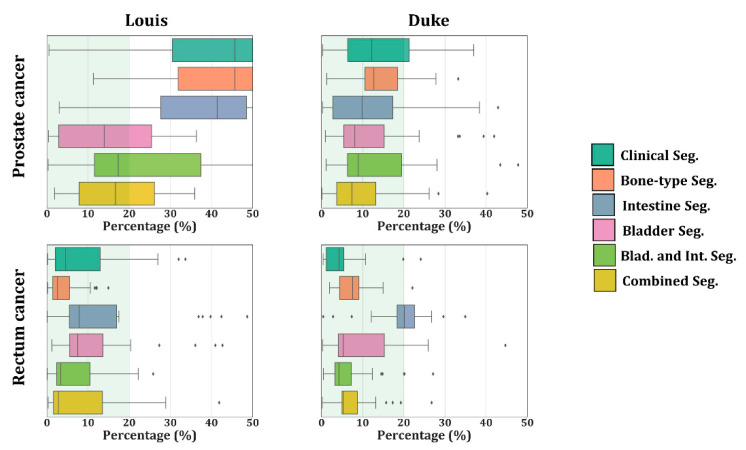
Boxplot with the maximum |dRD| in the male models. |dRD| was normalized to the maximum SAR for each tissue in the benchmark and expressed as a percentage (%). The inter-quartile range represents the middle 50% of the dataset, where the right box line represents 75% of the dataset that falls below the upper quartile, the left line represents 25% of the dataset that falls below the lower quartile, and finally, the middle line represents the median. The line extending from the box represents 2.5% and 97.5% limits of the dataset.

Figure 5 and Figure 6 present the absolute difference in RD (|dRD|) between the detailed benchmark and the segmentations under study for each tissue in female and male virtual models. Figure 5 indicates that hotspots were not correctly predicted for less detailed segmentation in female models. Overall the best results were obtained when intestines were included in the segmentation (median |dRD| < 20%). For cervical cancer, segmentations that included the delineation of intestines improved |dRD| significantly. For rectum cancer, median |dRD|’s were close to zero for Ella, but higher |dRD| median values were observed for Billie and Yoon Sun. In general, segmentations with intestines included (intestine and combined segmentation) performed best in terms of mimicking the average SAR of the detailed segmentation.

For the male models, the best results were obtained when the bladder wall and urine were included in the segmentation as a separate organ (bladder segmentation; bladder and intestine segmentation; and combined segmentation) since the median |dRD|’s were close to zero. In the exception of Louis and a prostate target, median |dRD| was always below 10%. The results for the Louis model showed the highest variation in |dRD|, but the highest |dRD| median was reduced from >40% for the three segmentations (Clinic, Bone-type, and Intestine Seg.) to below 20% when the bladder was segmented.

## 4. Discussion

### 4.1. Study Results

This study illustrates that the list of tissues selected for segmentation affects the predicted hyperthermia treatment quality. Tissue segmentation combined with high dielectric tissue property contrasts influences the predicted SAR by a maximum of 20% in |dTHQ|. As presented in Table 3, the addition of the intestines led to the lowest |dTHQ| (~5%) for female models. This stems from the electric conductivity of the intestines that, on average, was 30% higher when compared to muscle. For rectum tumors in female models, the inclusion of high electric conductivity intestines at the center of the body resulted in higher energy absorption of the E-field coming from the posterior side, which translated into an average |dRD| of 16% and a |dTHQ| of 8%. In cervix cancer, the small and highly conductive structures, i.e., urethra and nerve, were close to the target region and, therefore, have a substantial effect on the SAR distribution. The impact of the intestines for the Yoon-Sun and Billie models were different than in Ella due to the higher volume percentage of these tissue structures around the target. In Ella, the discrimination of the different bone structures improved the THQ prediction due to the large bone structures (Table 3). For males, Table 4 shows that delineation of the bladder, and inclusion of the dielectric contrast due to bladder filling (urine), improved the THQ prediction, leading in general to a THQ variation lower than 5%, i.e., an expected temperature change ≤0.2 °C [32]. In Louis, the inaccuracy without the bladder segmentation, however, was more than 20%. The inclusion of high organs’ volume with high conductivity showed it to have a high impact on the accuracy of the predicted SAR. This can be observed from Table 2 and Figure 2 [39], where models including a larger size of bladder and bladder lumen (urine) with high dielectric properties [38,43] presented high impact in predicted SAR (Table 4 and Figure 6). Overall, the results showed that delineation of the intestines (in females) and the bladder and bladder–lumen (in males) provide valuable additions to the clinical protocol (muscle–fat–bone–air) for HTP. These observations are based on the virtual model anatomies. Further, the limitations of the delineation of these tissue in clinical settings are discussed.

Looking at the SAR patterns expressed in |dRD|, delineation of the intestines for the female models and each cancer type led to values lower than 20% in most of the tissues. For rectum cancer, Figure 5 shows that the median |dRD| was lower or approximately equal to 10% when the intestines were included in the segmentation as a separate organ. As expected, the same distributions also showed that each increase in segmentation detail contributed to lowering the SAR differences with respect to the detailed patient model. As shown by Figure 6, for the male models and each cancer type, |dRD| was consistently low when the bladder was delineated. In general, the |dRD| results confirmed the THQ based analysis that adding intestines in females improves the results and the THQ finding that adding the bladder and bladder–lumen improved. Significantly, the results for males were partially confirmed since |dRD| was not always lower than 10%.

### 4.2. Clinical Translation

In this study, we used models of healthy volunteers to predict the impact of tissue segmentations in patients. Therefore, we started this study by investigating the representative value of such models. In this respect, Billy and Louis had the largest variations in the evaluation parameters since they were thinner models in which the total volume inside the hyperthermia applicator was lower than Ella, Duke, and Yoon Sun. As shown by Figure 2, structures, such as the reproductive organs, kidney, liver, and intestines, have a relatively higher relative volume in Billie and Louis. The related high electric conductivity of these regions translates into higher SAR values. These high differences are evidence of the important impact of the combination of segmentation and dielectric properties on the THQ prediction [18,34,38,43]. Further, the regression line between fat and muscle was similar in the Virtual Family models, as compared to the regression line for the models of treated patients. However, since the Virtual Family volunteers were, on average, younger than the treated patients, they have a higher relative muscle volume [49], which may result in slight differences in the energy penetration. Earlier studies reported higher rates of subcutaneous tissue toxicity when patients had thicker dorsal subcutaneous fat [10], and that the thickness of subcutaneous fat is correlated with lower achievable heating times in bladder cancer [50]. However, since the fat–muscle correlation for volunteers followed the same trend as the results in patients and span the same fat–muscle percentages, we expect that these differences do not affect our results based on comparisons of relative differences in SAR.

Although the result of the current study indicates the importance of including a more detailed tissue segmentation in HTP, the study faces the common weakness or difficulty of defining the most predictive and sensitive parameter correlating with HTP quality and treatment outcome [10,12]. Hyperthermia outcome, as stated previously, has been correlated in clinical treatment only to the measured thermal dose that was applied. Furthermore, in theoretical modeling, a linear correlation between THQ and the simulated steady-state temperature was observed. Though, there are still no studies that correlate any predicted hyperthermia quality parameter (SAR, aSAR, THQ, etc.) with treatment outcome or thermal dose. Hence, in the absence of such a correlation between THQ and treatment outcome, we hypothesized a threshold for clinical relevance based on the study made by Canters et al., knowing that this study was confounded by the uncertainties of thermal tissue properties. The other parameter used to evaluate the impact of delineated specific tissues was the maximum averaged-tissue SAR. For simulated SAR, Paulides et al. presented a linear relation between simulated SAR and measured temperatures in the head and neck region [34]. Hence, this parameter was assumed to indicate the same observations for pelvic hyperthermia. This was used to quantify the tissue dose in models where more tissues were delineated.

As mentioned before, with regard to this particular study, we based most of the clinical impact assumptions on theoretical and simulation studies. A point of concern, however, is whether the results are predictive for the clinical practice. The virtual models were based on healthy volunteers, where an alternative for this study would have been to investigate the effect of detailed modeling in a patient group using MR images of patients. Unfortunately, a detailed assessment of the anatomy was too demanding for cancer patients due to the long scanning time it takes to make such images for a sufficiently large body model. We verified the muscle–fat percentages and, therefore, consider the models sufficiently predictive for this analysis, including only relative metrics. In summary, although based only on a simulation study and not validated using measurements, we showed that the tissue list was one of the issues that can be considered when improving current HTP.

For further conclusions, other limitations of this study should be noted besides those aforementioned. The uncertainty of our results depends on the reliability of the literature values of the electromagnetic tissue parameters. The dielectric properties used in this study were all taken from the literature [38], where human tissue and in vivo measurements were selected in preference over animal tissue and in vitro measurements. Another limitation is that we performed our analysis in only five models in a straight position. Although the models were not in the ideal treatment position, i.e., placed in the hammock within the Sigma Eye, we do assume that this effect was neutralized by the fact that we made only relative comparisons. At the same time, newly developed systems for deep hyperthermia, such as the ALBA-4D (Alba Hyperthermia, Rome, Italy) [4,51] and the Universal MR-compatible Sigma Eye applicator (Pyrexar Medical Corp., Salt Lake City, UT, USA) [52,53], do provide a flat table patient positioning. Beside the straight position, we assumed a treatment scenario with models lying down motionless, which is very unlikely to occur in a realistic treatment. The SAR peak locations and values depend on the actual posture of the model. Hence, the maximum SAR location might change as a function of time for moving subjects, conducting a lower-time average SAR [54]. A drawback of these models that follow the limitations mentioned before is the non-real-time patient anatomy. As mentioned before, at Erasmus MC, a bladder catheter is kept open during the treatment to have an empty bladder. According to our clinical procedure, the bladder from these models was not representative. However, for other hyperthermia treatment procedures, a filled bladder can be representative of hyperthermia treatment. In addition, the virtual models do not include gastrointestinal air and its motion. Finally, another limitation of this study is that, although we used realistically shaped tumors, they remain artificial.

## 5. Conclusions

In this study, we investigated the impact of segmentation detail on the SAR predictions for deep hyperthermia in the pelvic region. Acting as surrogates for thermal dose, differences in predicted |dTHQ| and |dRD|. were analyzed as metrics for hyperthermia treatment quality and the overall SAR distribution. As expected, the position and percentage volume of high dielectric tissues around the target area influenced the SAR distributions. Therefore, the main finding of this study is that the inclusion of tissues with high water content, such as the bladder and intestines, may result in a clinically relevant impact on the SAR distribution and on the predicted hyperthermia treatment quality according to our pre-established thresholds.

We found that the inclusion of the intestines to our clinical protocol (muscle–fat–bone–air) improves the |dRD| predictions for females between 5 to 10%. However, in |dTHQ| no significant improvement was observed. For male models, additional segmented structures led to a low improvement in |dTHQ| and an inconsistent improvement in |dRD|. Our results underline our current clinical segmentation protocol and provide directions to prioritize improvements, e.g., in cases where bladder volume in patients strongly changes between the imaging for HTP and the hyperthermia treatment.

## Figures and Tables

**Table 1:** Tissues delineated in each segmentation.

Segmentation List Name	Delineated Tissues
Detailed	80 tissues and GTV
Clinical	fat, muscle, cortical bone, and GTV
Bone-type	fat, muscle, cortical bone, GTV, bone marrow, and cancellous bone
Bladder	fat, muscle, cortical bone, GTV, urine, and bladder wall
Intestine	fat, muscle, cortical bone, GTV, small intestine wall, small intestine lumen, large intestine wall, large intestine lumen
Bladder and intestine	fat, muscle, cortical bone, GTV, urine, bladder wall, small intestine wall, small intestine lumen, large intestine wall, and large intestine lumen
Combined	fat, muscle, cortical bone, GTV, bone marrow, cancellous bone, urine, bladder wall, small intestine wall, small intestine lumen, large intestine wall, and large intestine lumen

**Table 2:** Relative permittivity ($\varepsilon_{r}$), effective conductivity ($\sigma_{\mathrm{eff}}),$ and mass density ρ for the selected tissues at 100 MHz.

Tissue	$\varepsilon_{r}\;(-)$	$\sigma_{\mathrm{eff}}\;(S/m)$	$\rho \;({\mathrm{kg}/m}^{3})$
Applicator shell	2.0	0.004	1180
Water (water bolus)	80.9	0.002	1000
Fat	12.7	0.007	911
Muscle	66.0	0.708	1090
Bone (cortical)	15.3	0.006	1908
Bone (marrow)	14.3	0.159	1029
Bone (cancellous)	27.6	0.173	1178
Small intestine wall	96.5	1.660	1030
Small intestine lumen	80.0	2.000	1000
Large intestine wall	81.8	0.680	1088
Large intestine lumen	66.0	0.708	1090
Bladder wall	22.7	0.294	1086
Urine	49.9	1.750	1024
Tumor (GTV)	70.0	0.750	1050

**Table 3:** The impact of different segmentation on the simulated hyperthermia dose within females. This was quantified in the absolute value of the relative difference in THQ (|dTHQ|). Note that the evaluation for each model and segmentation was the maximum between the three different sizes. |dTHQ| values approximately equal and below 5%, i.e., the threshold for clinical relevance (~0.2 °C), are given in boldface. For each cancer type and model, maximum values and their standard deviation are listed.

Segmentation	Cervix Cancer			Rectum Cancer		
	Billie	Ella	Yoon-Sun	Billie	Ella	Yoon-Sun
Clinic	22.8 ± 7.2	15.0 ± 3.9	7.0 ± 0.6	9.9 ± 3.1	18.7 ± 1.3	8.2 ± 1.6
Bone-type	21.1 ± 5.6	11.3 ± 5.8	5.6 ± 0.3	10.3 ± 2.0	18.1 ± 4.1	5.8 ± 0.1
Bladder	23.8 ± 7.4	20.8 ± 9.6	10.2 ± 1.3	13.0 ± 2.6	16.7 ± 2.7	7.4 ± 1.0
Intestine	**5.3** ± 2.4	12.2 ± 5.2	11.8 ± 4.3	5.8 ± 2.2	13.2 ± 2.8	**5.2** ± 0.3
Blad. and Int.	8.1 ± 3.5	10.2 ± 3.2	18.0 ± 7.7	14.0 ± 6.6	15.1 ± 3.0	5.9 ± 1.1
Combined	**3.4** ± 0.1	7.8 ± 3.5	8.2 ± 3.0	**0.7** ± 0.3	14.5 ± 3.0	**4.7** ± 0.9

**Table 4:** The impact of different segmentation on the simulated hyperthermia dose within males. This was quantified in the absolute value of the relative difference in THQ (|dTHQ|). Note that the evaluation for each model and segmentation was the maximum between the three different sizes. |dTHQ| values approximately equal and below 5%, i.e., the threshold for clinical relevance (~0.2 °C), are given in boldface. For each cancer type and model, maximum values and their standard deviation are listed.

Segmentation	Prostate Cancer		Rectum Cancer	
	Louis	Duke	Louis	Duke
Clinic	20.6 ± 0.6	**4.4** ± 1.3	**5.0** ± 1.6	**3.1** ± 0.5
Bone-type	21.6 ± 2.4	**4.7** ± 1.3	**3.7** ± 1.2	7.6 ± 2.4
Bladder	**3.6** ± 0.8	**2.2** ± 0.2	**0.7** ± 0.2	**2.9** ± 2.3
Intestine	22.1 ± 2.0	6.2 ± 1.4	**3.2** ± 0.8	**2.6** ± 1.2
Blad. and Int.	14.2 ± 3.9	**2.4** ± 0.8	**1.2** ± 0.6	**1.8** ± 0.9
Combined	8.0 ± 3.3	**2.1** ± 0.9	**1.3** ± 0.6	**1.9** ± 0.6
